# Efficiency of the cytochrome c oxidase subunit II gene for the delimitation of termite species (Blattodea: Isoptera) in the state of Paraíba, northeastern Brazil

**DOI:** 10.1371/journal.pone.0328685

**Published:** 2025-09-10

**Authors:** Sara Rikeley Paulino Monteiro, Antonio Carvalho, Renan Rodrigues Ferreira, Rozzanna Esther C. R. Figueirêdo, Alexandre Vasconcellos, Ricardo Koroiva

**Affiliations:** 1 Laboratório de Termitologia, Departamento de Sistemática e Ecologia, Centro de Ciências Exatas e da Natureza, Universidade Federal da Paraíba, João Pessoa, Paraíba, Brazil; 2 Instituto de Ciências Biológicas, Universidade Federal do Pará, Belém, Pará, Brazil; 3 Departamento de Engenharia e Meio Ambiente, Universidade Federal da Paraíba, Rio Tinto, Paraíba, Brazil; National Cheng Kung University, TAIWAN

## Abstract

With the aim of expanding the possibilities of identifying termite species, in the present study we generated genetic data based on sequences of the mitochondrial gene encoding *cytochrome c oxidase subunit II* (COII) for termites (Blattodea: Isoptera) occurring in the state of Paraíba, northeastern Brazil. The genetic data were obtained from 135 COII sequences identified in 28 genera and 48 species. These are the first COII sequences for 15 taxa (31.2%) available in public sources. Using delimitation methods based on distance (ASAP and ABGD) and tree (GMYC, bPTP, mPTP and PTP), we confirmed the efficiency of this technique in delimiting most species. However, the assessment of intraspecific and interspecific variation revealed the occurrence of species with intraspecific genetic variation classified as high (> 2%). The analysis of identification efficiency based on our genetic data revealed a high rate of correct identifications (91.80% to 100%), confirming the efficiency of COII in species identification. The generation of these genetic data contributed as an alternative method for future identifications, allowed the understanding of the phylogenetic diversity of some termite species in Paraíba and the application of new molecular techniques to collect data on the conservation of the state.

## Introduction

Termites are eusocial insects of the order Blattodea [1], organized into specific castes, each with different morphological and physiological characteristics related to the different functions they perform in the colony [2]. There are 2,994 species of living termites [3], with great diversity and biomass in tropical regions [4–6]. In Brazil, at least 350 species of termites have been identified, classified into five families: Kalotermitidae, Heterotermitidae, Rhinotermitidae, Serritermitidae and Termitidae [3].

Usually, external and/or internal morphological characters are used to determine the taxa of these eusocial insects, with the soldier caste containing the most useful characters for taxonomy [7,8]. However, it is worth noting that the presence of soldiers is not observed in all termite species. This is the case of the species of the subfamily Apicotermitinae, which occur in the Neotropical region. Due to this characteristic, the delimitation of genera or species in this group is mainly based on the worker caste, with the internal characters being the most analyzed [9], which complicates taxonomic work and leads to a knowledge gap about the diversity of Apicotermitinae in the Neotropical region [10].

Although traditional identification is applied to species that have soldiers, the morphological approach has some limitations in certain situations due to intraspecific variation, old descriptions with sparse diagnoses and the presence of cryptic species [11–13]. In order to reduce the existing limitations when only morphological characters are used in the description and identification of termite species, some authors have started to analyze other parameters besides morphology [14–17], which has led to an increase in information to distinguish species. One of these parameters is molecular characters, which were originally used more in studies focused on understanding phylogeny or phylogeographic relationships [18–21]. In recent years, however, these traits have proven useful for species identification and delimitation, with DNA barcoding being an example of the methods used to achieve these results [22–24].

In termites, the mitochondrial gene (mtDNA) encoding *cytochrome c oxidase subunit II* (COII) has been shown to be an efficient gene for species delimitation and identification. This polymorphic gene [25,26] can be easily amplified in termites [26] and is recommended for the use of DNA barcoding in soil-feeding termites and non-soldier caste specimens [10,14]. However, in order for the analysis of COII sequences to be used for species identification, genetic data must be generated and deposited in public repositories so that sequence comparisons can be made in the future with already known and deposited information [27–29]. There are numerous efforts in the literature to create a database for insects [30–34].

Aware that the use of exclusively morphological traits in termite identification has its limitations, and considering that most genetic data refer to groups with greater charismatic attraction [29], especially in easily accessible locations [35], the present study aims to generate genetic data using the mitochondrial COII gene for termite species occurring in the state of Paraíba. This state is located in an area of Brazil that is poorly explored in terms of genetic data generation and currently has 78 species (Vasconcellos & Chaves 2024, Alexandre Vasconcellos; Rozzanna Esther Cavalcanti Reis de Figueirêdo Chaves, 2024, “Biodiversidade de Cupins da Paraíba, Brasil”, https://doi.org/10.48472/DATAPB/Z0VB1K, DATAPB, V1). We also test the efficiency of the COII gene in delimiting the termite species present in this region. Finally, the generation of these genetic data will allow the acquisition of molecular characteristics that will help in the identification of the termites present in this region.

## Materials e methods

### Data collection

**Fig 1:**
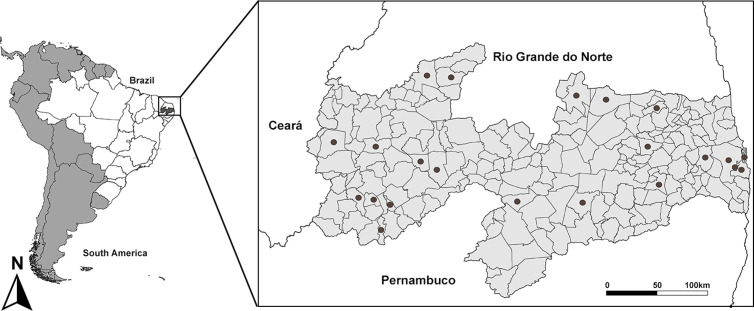
Municipalities where samples were collected for the COII termite sequence genetic data in the state of Paraíba, Brazil. The information about the collection sites and other relevant details about the samples can be found in Table S1. The map was created with QGIS 3.38.2.

To generate this genetic data, 238 specimens were used and assigned to 64 morphological species. The specimens came from collections in different municipalities of the state of Paraíba (Fig 1), located in northeastern Brazil between latitudes 6º02’12” and 8º19’18” S and longitudes 34º45’54” and 38º45 ‘45” W (see Table S1), and were deposited in the Isoptera collection of the Federal University of Paraíba, Brazil. The collection was approved by the Brazilian Institute of Wildlife and Environment/Ministry of Environment (SISBIO license number 72602−4).

The identification of the collected specimens was based on morphological characteristics using identification keys [8,36] and species descriptions of termites from the literature. In addition, comparisons were made with previously identified specimens deposited in the Isoptera collection of the UFPB. Most identifications were made using individuals of the soldier caste; however, for species belonging to the subfamily Apicotermitinae, individuals of the worker caste were used.

The state’s vegetation varies throughout its territory, with Caatinga being the predominant biome, but Atlantic Forest and coastal forests are also present. According to the Köppen classification, the region comprises different climate zones: tropical with a dry winter season (Aw), humid tropical (Am), tropical with a dry season (As), and semi-arid (Bsh) [377]. The amount of precipitation varies between 300 mm and 1900 mm at a temperature of around 24°C [37].

### Amplifying and sequencing

For DNA extraction, the heads of specimens from the soldier and worker castes were used to reduce the risk of contamination with the genetic material of the symbionts in the termite gut [15]. The Blood & Tissue DNA Mini Kit (Ludwig Biotec, Alvorada, Brazil) was used for the extraction, following the protocol suggested by the manufacturer. The primers Mod – A-Tleu (5’ CAG ATA AGT GCA TTG GAT TT 3’) and B- tLys (5’ GTT TAA GAG ACC AGT ACT TG 3’) were used to amplify the region of the mitochondrial gene encoding *cytochrome c oxidase subunit II* (COII) [38,39]. Polymerase chain reaction (PCR) amplification was performed according to the following protocol: 5.0 μL 1 × Colorless GoTaq® Flexi Buffer (Promega Corp., Madison, WI); 5.0 μL 0.2 mM dNTP; 5.0 μL of each primer at 0.2 mM (forward and reverse); 3.0 μL 2 mM MgCl2; 0.2 μL 1U Taq polymerase (Promega Corp., Madison, WI); and 2 μL extracted DNA, for a total volume of 25 μL. The amplification cycle included an initial denaturation of 2 minutes at 94°C, annealing of 45 seconds at 47°C, an extension of 1 minute at 72°C and a final extension of 10 minutes at 72°C. The success of amplification was assessed by electrophoresis in a 2% agarose gel using Safer, a non-mutagenic dye, and visualized under ultraviolet light. Successful PCR products were bidirectionally sequenced using an ABI 3130 Genetic Analyzer (Applied Biosystems).

### Analysis of the data

Analyzes to check the quality of the sequences obtained and for editing and assembly, if required, were performed using GENEIOUS v 9.1.3 software [40]. Alignment was performed in the same software using the MAFFT v.7.017 [41] module in the default configuration. To confirm the absence of contaminants, a BLAST was performed on the NCBI website (NCBI- https://blast.ncbi.nlm.nih.gov/Blast.cgi, accessed May 10, 2023). To complete the database, COII sequences from termites collected in Paraíba that were already available in the GenBank repository, were also added (see Table S2).

### Species delimitation

For the analyzes, the species were grouped by family, as various studies aimed at understanding the evolutionary processes within the infraorder Isoptera have shown that the families diverged millions of years ago [42–44]. In addition, the most recent classification for termites based on molecular data [45] was used. Two approaches were used to delimit species: Distance and Tree [46]. In the distance method, we performed the automatic analysis barcode gap discovery (ABGD) [47] online (https://bioinfo.mnhn.fr/abi/public/abgd/abgdweb.html; accessed June 20, 2023) with the Kimura-2 parameter (K2P) distance model [48] and the following settings: Pmim = 0.001; Pmax = 0.2; Step = 20; Nb bins = 20, and the relative gap width (x) = 0.5. We also run the Assemble Species by Automatic Partitioning (ASAP) analysis [49] online (https://bioinfo.mnhn.fr/abi/public/asap/; accessed June 20, 2023) with the same model and default settings. In ASAP, only the partition that had the lowest asap score value was considered (asap score of Kalotermitidae = 2.00, Heterotermitidae = 1.50 and Termitidae = 5.00).

When delimiting species based on phylogenetic trees, we performed the following analyzes: Poisson Tree Process [50] (mPTP, PTP and bPTP) and Generalized Mixed Yule Coalescent (GMYC) [51,52]. We have evaluated the best nucleotide substitution model for the tree method delineation using the XSEDE JmodelTest2 program [53,54], on the CIPRES v. portal. 3.3 [55] (https://www.phylo.org/portal2/login!input.action; accessed on June 20, 2023). The result obtained for the three families was the GTR + I + G model.

The mPTP [56] was performed online (https://mptp.h-its.org/#/tree; accessed June 20, 2023), using the default settings and removing sequences that belonged to the outgroup. For the other analyzes, PTP, bPTP and GMYC, iTaxo Tools software was used [46]. The ultrametric tree used in the GMYC analysis was generated using the program BEAST v1.10.4 [57] with the following parameters: relaxed clock with lognormal distribution, the Yule process speciation model, GTR + I + G and constant population size. The number of generations used in the CIPRES v. environment. 3.3 [55] varied depending on the family analyzed (Kalotermitidae = 2.5 x 10⁸ generations, Heterotermitidae = 2 x 10⁸ generations, Termitidae = 3.5 x 10⁸ generations). The program Tracer v1.7.2 [58] was used to evaluate the results based on ESS values that were considered satisfactory (ESS > 200). In addition, the program TreeAnnotator v1.10.4 [57] was used to create a consensus tree, excluding the first 10% as “burn-in”.

To represent the phylogenies, we created trees with the maximum likelihood method using the IQ- Tree web server [59] (http://iqtree.cibiv.univie.ac.at; accessed June 20, 2023)The trees were visualized with the software Figtree v1.4.4. Sequences from GenBank were used as an external group in all analyzes. However, a different external group was used for each family analyzed in this work (see Table S2).

### Intra and interspecific divergence

MEGA11 software [60] was used to assess intraspecific and interspecific divergence (mean and maximum intraspecific variation and minimum distance to nearest neighbor species) for the sequenced COII gene sequences, using the Kimura 2-parameter (K2P) distance model [48] with the settings provided by the program. This distance model was chosen because previous studies have used it to calculate genetic variation. Despite potential problems with thresholds, we opted for the default threshold of 2%, which is commonly cited in DNA barcoding studies [61]. This analysis included all three families together.

### Specimen identification

The success of the identification of termites from the state of Paraíba using the COII gene was verified using the Best Match (BM), Best Close Match (BCM), and BOLD Identification Criterion (BIC) using the Species Identity and Evolution package (SPIDER v. 1.3) [62] in program R [63]. Performing these analyzes involves a sequence-based identification simulation in which each sequence is treated as an unknown specimen, and our data library is used to assign a species-level identification. No singletons were used in the queries, and all analyzes were performed using the K2P model.

In this study, we analyzed distances in four different scenarios: (i) 1%, as defined by the BOLD system [64]; (ii) 2%, as proposed by Hebert, Ratnasingham, and de Waard (2003), and commonly used as a threshold for insect DNA barcoding [65]; (iii) a percentage determined by the “localMinima” function in SPIDER, which calculates genetic distance based on the data [62]; and (iv) a percentage determined by the “ ThreshVal “ function, also included in SPIDER, which calculates the genetic interval with the smallest cumulative error [62].

## Results

### Sampling and data set

Of the 64 species originally considered for inclusion in the genetic data, sequences were obtained for 48 species from the state of Paraíba. The genetic data contain 135 sequences, with an average of 2.8 specimens per species. However, it should be noted that these genetic data contain five singletons, for species such as *Cryptotermes brevis* (Walker, 1853), *Ibitermes inflatus* Vasconcellos, 2002, *Nasutitermes callimorphus* Mathews, 1977, *Neotermes hirtellus* (Silvestri, 1901) and *Tauritermes bandeirai* Scheffrahn & Vasconcellos, 2020. The analyzed sequences had a length of 601 base pairs for Heterotermitidae, 610 base pairs for Kalotermitidae, and 605 base pairs for Termitidae. All sequences were deposited (Table S1), and the first COII sequence of 15 taxa (31.2%) was deposited in the GenBank repository.

### Species delimitation

**Fig 2:**
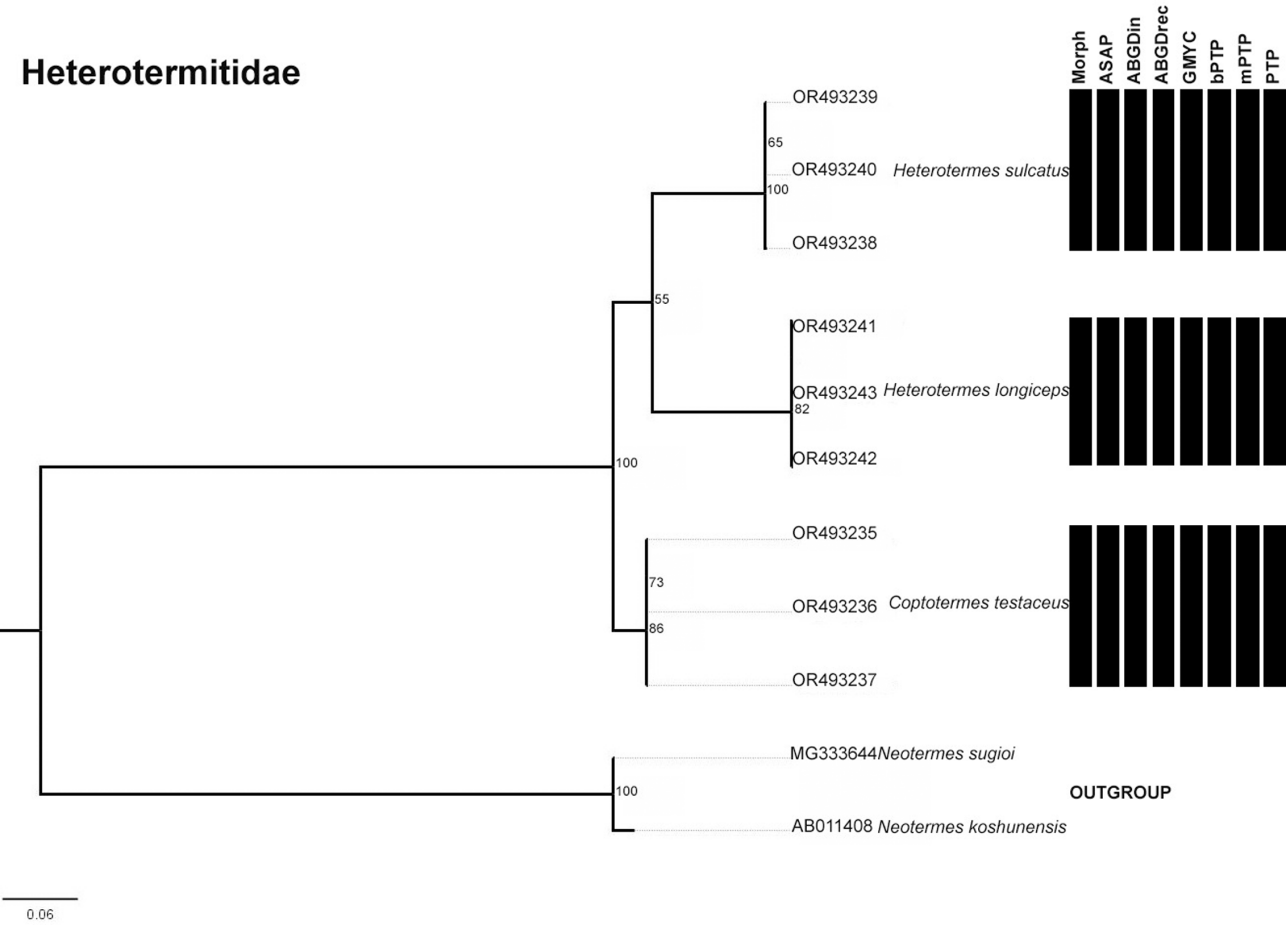
Maximum likelihood (ML) trees of termites generated with IQ-Tree based on sequences of species from the family Heterotermitidae collected in the state of Paraíba. The numbers represent the bootstrap values of the nodes. Each bar represents a specific delimitation method: Morphological, ASAP (Assemble Species by Automatic Partitioning), ABGDin (Automatic Barcode Gap Discovery: initial partition), ABGDrec (Automatic Barcode Gap Discovery: recursive partition), GMYC (Generalized Mixed Yule Coalescent), bPTP (Bayesian Poisson Tree Process), mPTP (multi-rate Poisson Tree Process) and PTP (Poisson Tree Process); gray colored bars show divergences to the morphological delimitation.

**Fig 3:**
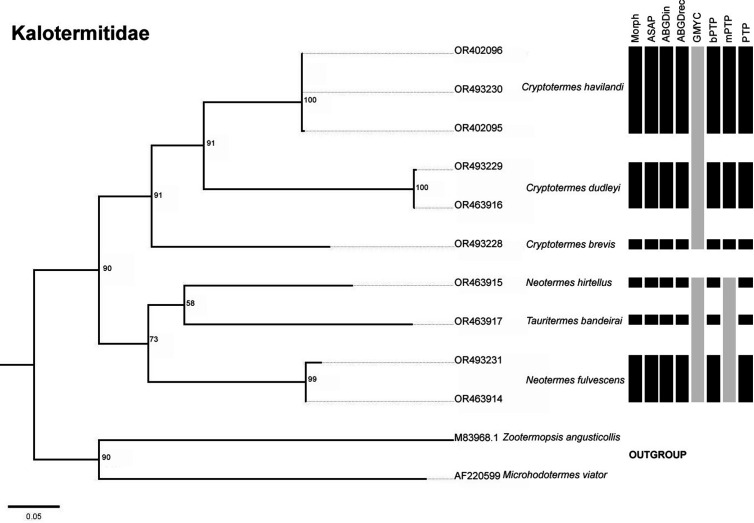
Maximum likelihood (ML) trees of termites generated with IQ-Tree based on sequences of species from the family Kalotermitidae collected in the state of Paraíba. The numbers represent the bootstrap values of the nodes. Each bar represents a specific delimitation method: Morphological, ASAP (Assemble Species by Automatic Partitioning), ABGDin (Automatic Barcode Gap Discovery: initial partition), ABGDrec (Automatic Barcode Gap Discovery: recursive partition), GMYC (Generalized Mixed Yule Coalescent), bPTP (Bayesian Poisson Tree Process), mPTP (multi-rate Poisson Tree Process) and PTP (Poisson Tree Process); gray colored bars show divergences to the morphological delimitation.

**Fig 4:**
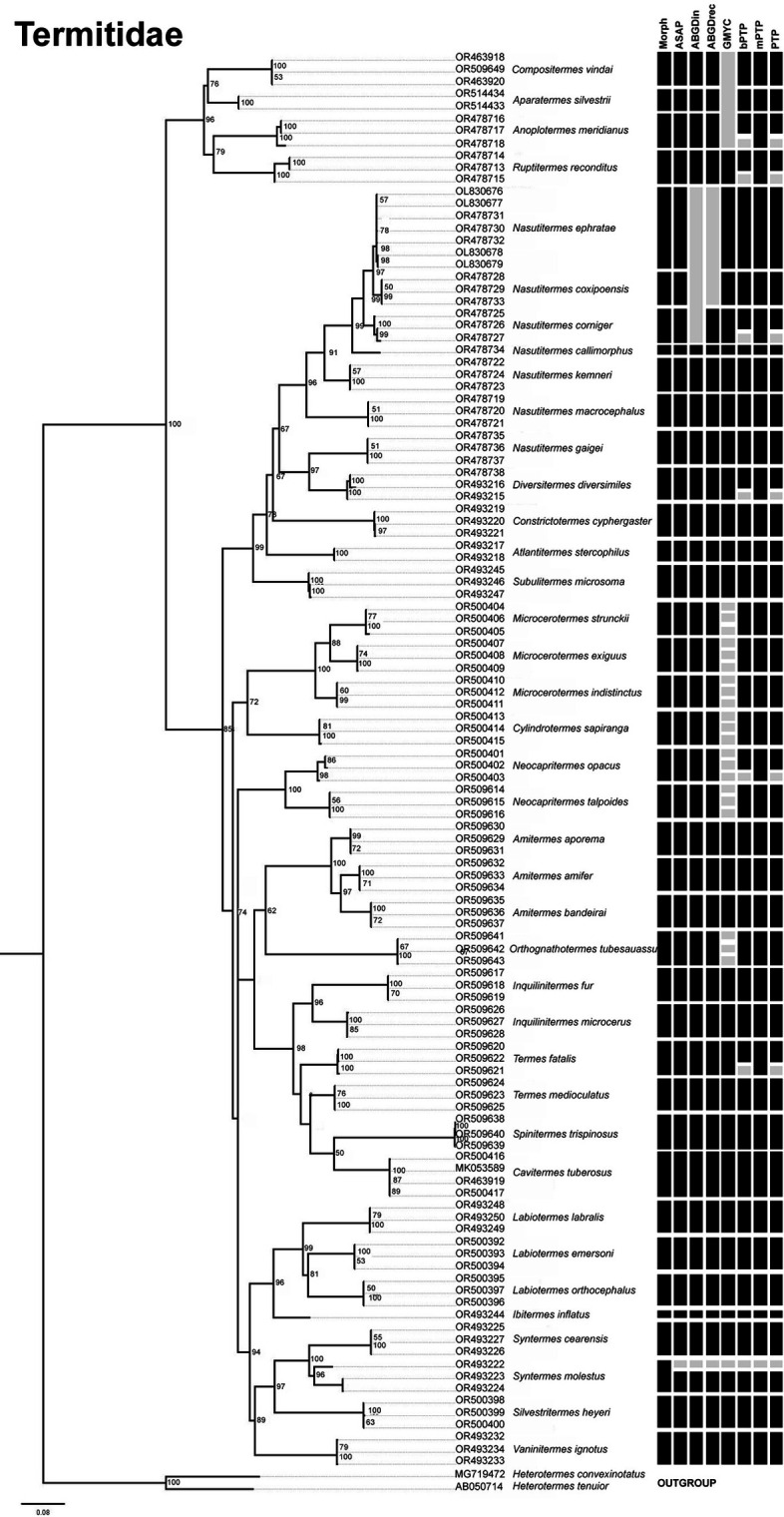
Maximum likelihood (ML) trees of termites generated with IQ-Tree based on sequences of species from the Termitidae family collected in the state of Paraíba. The numbers represent the bootstrap values of the nodes. Each bar represents a specific delimitation method: Morphological, ASAP (Assemble Species by Automatic Partitioning), ABGDin (Automatic Barcode Gap Discovery: initial partition), ABGDrec (Automatic Barcode Gap Discovery: recursive partition), GMYC (Generalized Mixed Yule Coalescent), bPTP (Bayesian Poisson Tree Process), mPTP (multi-rate Poisson Tree Process) and PTP (Poisson Tree Process); gray colored bars show the divergence to the morphological delimitation.

The number of possible species varied depending on the delimitation method used. Based on the morphological approach, the following number of species was considered for each family: six species for the Kalotermitidae, three for the Heterotermitidae, and 39 for the Termitidae. However, the Heterotermitidae was the only family in which all molecular delimitations matched the morphological delimitations (Fig 2). In the Kalotermitidae, there were only two methods that did not agree with the morphological delimitation: mPTP, which considered four possible species, and GMYC, which combined two possible species (Fig 3). In the case of Termitidae, the delimitations resulted in different values: 40 for ASAP, ABGD- initial partition in 38, ABGD- recursive partition in 39, GMYC in 52, bPTP in 46, mPTP in 40 and PTP in 46 (Fig 4).

### Intraspecific and interspecific divergence

**Fig 5:**
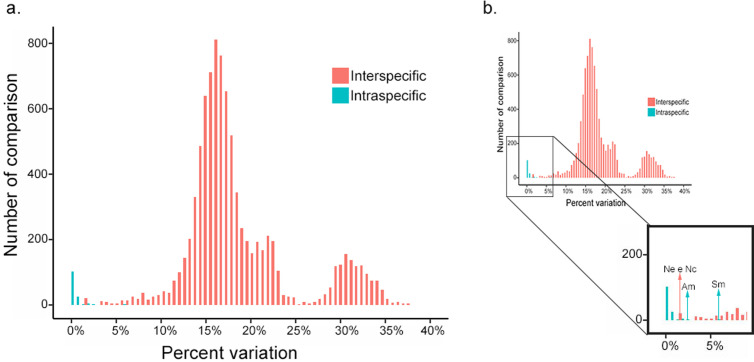
Recorded values of intra- and interspecific variations. **(a)** Intra- and interspecific divergence values found in all three families together; (b) focus on species that do not fit the given 2% pattern. Am (*Anoplotermes meridianus*), Nc (*Nasutitermes coxipoensis*), Ne (*Nasutitermes ephratae*) and Sm (*Syntermes molestus*).

Intraspecific genetic divergence ranged from 0% to 6.03% (Table S3), with a mean of 0.14% and a standard deviation of 0.80%. Interspecific genetic divergence ranged from 1.65% to 22.48% (Table S2), with a mean of 17% and a standard deviation of 6.02%. Some species did not meet the predetermined standard of 2%, such as *Syntermes molestus* (Burmeister, 1839) and *Anoplotermes meridianus* (Emerson, 1925), which had maximum intraspecific divergence values of 6.03% and 2.03%, respectively. In addition, species with low interspecific divergence were identified, namely *Nasutitermes ephratae* (Holmgren, 1910) and *Nasutitermes coxipoensis* (Holmgren, 1910) with 1.65% (as shown in Table S2). By plotting the intraspecific and interspecific variation values of the three families together, we were able to visualize the amplitude of the gap in the barcode based on the analyzed data (Fig 5a). It was found that the intraspecific and interspecific divergence values overlap (Fig 5b), which means that it is not possible to determine the barcoding gap for the constructed database.

### Specimen identification

When using SPIDER (Table 1) to test the feasibility of species identification from the genetic data, we were able to obtain a total of 130 correct identifications when using the BM method. The number of correct identifications using the BCM method ranged from 124 (limit of 1% and 1.06%) to 129 (limit of 2%). Finally, with the BIC method, the hit rate varied between 119 (limit of 2%) and 126 (limit of 1.55%).

**Table 1:** Summary of the results of the simulation of the identification of specimens with COII sequences. Three criteria were used to determine the effectiveness of specimen identification: Best Match (BM), Best Close Match (BCM) and Bold Identification Criterion (BIC). The numbers in parentheses indicate the number of identifications in each category, and the percentage was calculated for a total of 130 individuals. It is worth noting that singletons were not included in the analyzes.

Reference	Threshold value	Criterion	Correct	Incorrect	Ambiguous	No identification
Nearest-neighbor criterion	–	BM	100% (130)	0% (0)	0% (0)	0% (0)
Bold Threshold	1%	BCM	95.40% (124)	0% (0)	0% (0)	4.60% (6)
		BIC	95.40% (124)	0% (0)	0% (0)	4.60% (6)
Hebert, Ratnasingham & de Waard (2003)	2%	BCM	99.20% (129)	0% (0)	0% (0)	0.80% (1)
		BIC	91.50% (119)	0% (0)	7.70% (10)	0.80% (1)
“LocalMinima”	1.06%	BCM	95.40% (124)	0% (0)	0% (0)	4.60% (6)
		BIC	95.40% (124)	0% (0)	0% (0)	4.60% (6)
“ThreshVal”	1.55%	BCM	97.00% (126)	0% (0)	0% (0)	3.00% (4)
		BIC	97.00% (126)	0% (0)	0% (0)	3.00% (4)

## Discussion

The sequence encoding COII has been shown to be a gene that can be used to delimit termite species from the state of Paraiba, with most delimitations being consistent with identifications based on morphological characteristics. This type of result confirms what we found in the literature [66], but in the present study it was not possible to identify a barcode gap due to overlap between intraspecific and interspecific values. Applying an integrative approach using different methods to strengthen the reliability of taxonomic classification [67] and including at least three analyzes with different criteria for species delimitation [34], we found that only two species showed discrepancies in morphological identification: *Neocapritermes opacus* (Hagen, 1858) and *Syntermes molestus* (Fig 4).

**Fig 6:**
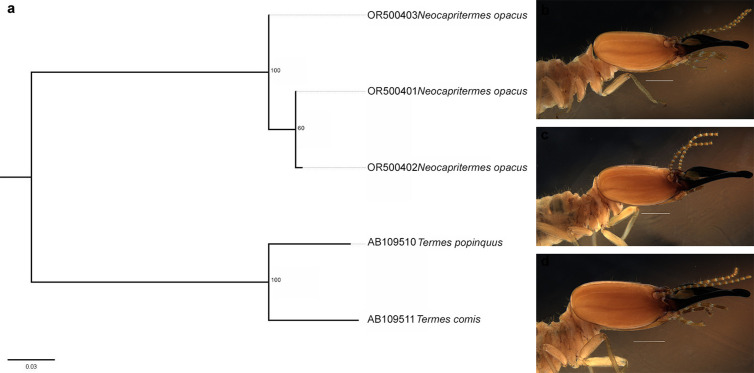
Relationship between the sequences of *Neocapritermes opacus.* **(a)** Maximum likelihood (ML) tree showing the relationship between the specimens used to obtain the *Neocapritermes opacus* sequences. The specimen coded OR500403 was delineated as a different species using the GMYC, bPTP and PTP methods. Photos of soldiers from the same batch from which the specimen was taken for sequencing; **(b)** OR500403; **(c)** OR500401; **(d)** OR500402. Photo by the author.

In the species *Neocapritermes opacus*, discrepancies were found in the delimitation by the tree method (GMYC, bPTP and PTP). Regarding the morphological characteristics of the species, it has already been mentioned in the literature that individuals differ in size [68]. Nevertheless, the specimens used to extract the sequences of this species exhibited a number of morphological features that can identify them as *Neocapritermes opacus*, with a labrum on the anterior side with three lobules, a pronotum almost twice as wide as long, and a moderately curved left mandible (Fig 6b-d).

**Fig 7:**
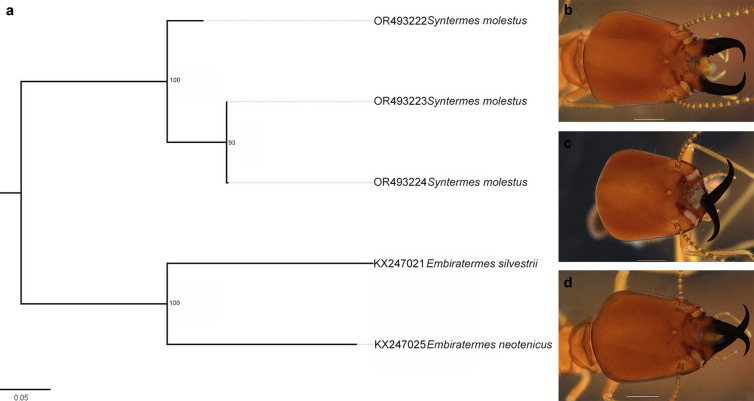
Relationship between the sequences of *Syntermes molestus.* **(a)** Maximum Likelihood (ML) tree showing the relationship between the specimens used to obtain the *Syntermes molestus* sequences. The specimen coded OR493222 was delineated as a different species in all methods. Photographs of soldiers from the same batch from which the specimen was taken for sequencing; **(b)** OR493222; **(c)** OR493223; **(d)** OR493224. Photo by the author.

High intraspecific variation was detected in the species *Syntermes molestus* (6.03%), which resulted in one of the sequences being classified as originating from a different species in all delimitations (Fig 4). However, the specimens used for DNA extraction showed morphological features belonging to the species in question, such as a very small or absent first marginal tooth of the right mandible, anteriorly converging sides of the head with strongly hooked mandibular tips, a posteriorly widening postmentum with only two hairs at the anterior corners, and a maximum width of the head greater than 2.9 [69] (Fig 7b-d). Based on the results obtained, we believe that there may be considerable intraspecific variation that requires a more detailed study of the populations of these animals.

Apart from *Syntermes molestus*, other results have shown that not all species considered in the compilation of this database meet the 2% standard (*Anoplotermes meridianus*, *Nasutitermes ephratae* and *Nasutitermes coxipoensis*), and this result is also found in the literature for other groups of invertebrates with the standard DNA barcoding gene, a mitochondrial gene encoding cytochrome c oxidase subunit I [70]. This type of result demonstrates that using a fixed value to delimit species in DNA barcoding does not cover the full range of evolutionary aspects that may exist in a community [34]. It also emphasizes the importance of calculating specific thresholds for each empirical dataset rather than relying on default values for species delimitation [47].

**Fig 8:**
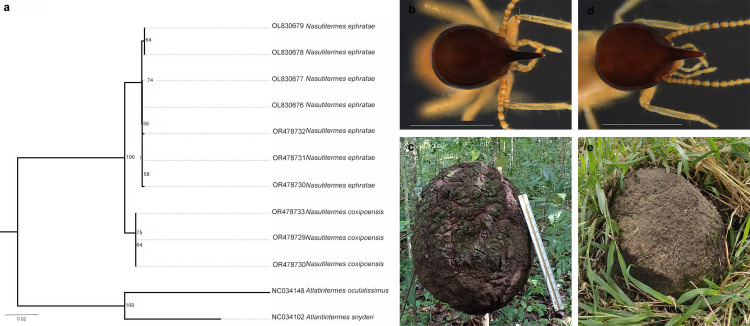
Sequence relationship of the species *Nasutitermes ephratae* and *Nasutitermes coxipoensis.* **(a)** Maximum Likelihood (ML) Tree showing the relationship between *N. ephratae* and *N. coxipoensis.* **(b)** Soldier of *N. ephratae;* **(c)** Nest of *N. ephratae;* **(d)** Soldier of *N. coxipoensis;* **(e)** Nest of *N. coxipoensis*. Photo by the author.

The species *N. ephratae* and *N. coxipoensis* were found to have an interspecific divergence of less than 2% (1.65%), which meant that the delimitation by the initial ABGD classification was recognized as a single species (Fig 4), as it is a method based on the distance between pairs and this low value of interspecific divergence affects the results [47]. Although these two species can be identified by the morphological characteristics of the soldiers [7,71], they pose a major challenge for accurate differentiation. This is because they are species with similar morphology [6666], which is why behavioral characteristics began to be recorded in addition to morphological characteristics to increase the reliability of identification. One of these obvious differences lies in the nests of the two species. *N. ephratae* builds arboreal nests (Fig 8c), which vary in color from light to dark. In addition, there is a reinforced chamber inside the nest in which the king and queen live [72]. In contrast, *N. coxipoensis* has epigeal nests (Fig 8e) with an irregular outer surface [73].

Regarding the morphology of the soldier caste of these species, *N. ephratae* has an elongate rather than broad head, with a nasus extending about half the width of the head [74–75] (Fig 8b). (Fig 8b). In contrast, in the soldiers of *N. coxipoensis*, the tip of the nasus is lighter in color, while the head is dark brown. The body, on the other hand, has a more yellowish color, which can vary to a dark brown. The pronotum of these soldiers takes the shape of a saddle, with an irregular anterior margin, and finally the abdominal tergites are smooth [71] (Fig 8d). The grouping of individuals of these species into a single species by one method and the low interspecific divergence emphasize the need for integrative taxonomy to provide a more reliable species delimitation [76].

**Fig 9:**
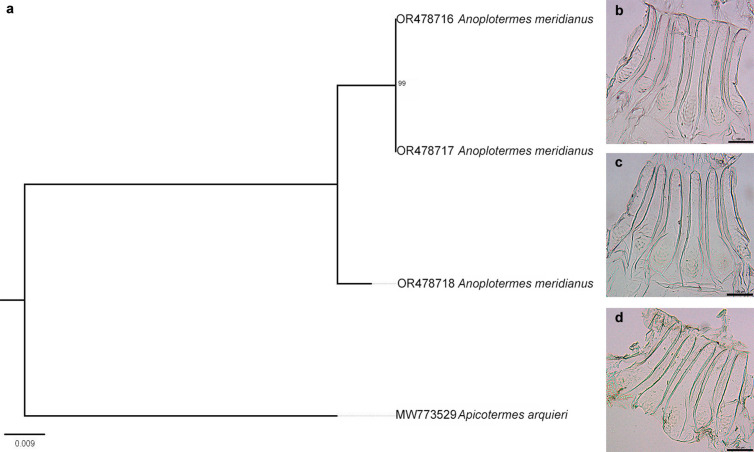
Relationship between the sequences of *Anoplotermes meridianus.* **(a)** Maximum likelihood (ML) tree showing the relationship between the specimens used to obtain the sequences of *Anoplotermes meridianus* with the specimen coded OR478718 delineated as a different species by bPTP and PTP. (bd) Photos of enteric valve from workers from the same batch from which the sample for sequencing was taken; **(b)** OR478716; **(c)** OR478717; **(d)** OR478718. Photo by the author.

Although *Anoplotermes meridianus* has an intraspecific divergence of more than 2% (2.03%), all sequences in all delimitations were determined to belong to a single species based on genetic distance. The specimens used for DNA extraction have features that identify them as *Anoplotermes meridianus*, such as the enteric valve unarmed, which has six regular cushions with rounded and smooth scales at the base (Fig 9b-d) [77]. Regarding the external morphology of the worker, an abdomen with a darker shade stands out due to its diet, while the antenna is composed of 14 articles, the third article being twice as large as the second [78].

There are some studies that attempt to expand the knowledge of the descriptive characteristics of the species of the genus *Anoplotermes* (Termitidae: Apicoterminae) [79,80]. However, these studies do not provide specific information on the description of *Anoplotermes meridianus*. Considering this gap, it is important to conduct review studies dedicated to this particular species to deepen our understanding of the characteristic traits that identify it and to clarify its relationship with other species within the genus, as *Anoplotermes meriadianus* has already been shown to be a possible sister group to *Humutermes* Bourguignon & Roisin, 2016 (Termitidae: Apicoterminae), demonstrating that the genus *Anoplotermes* is a paraphyletic group [81].

For genetic data to be used as a means of facilitating the identification of specimens, it is necessary to know whether it is possible to identify specimens to species level with it. In this regard, the number of correct identifications varied between 91.50% and 100%, with not a single incorrect identification, proving that it is possible to identify most termite species using the COII sequences. In comparison with other studies that have generated genetic data for different groups, we found similar results, e.g., for the genetic data of mosquitoes from French Guiana with a minimum hit rate of 98.7% [82], for the genetic data of Neotropical Odonata occurring in the Alto Prata basin with a minimum hit rate of 79% [33], and for the genetic data of butterflies from South America, which achieved a minimum accuracy of 95% [83].

The results of the best-match analysis showed a 100% identification success rate. However, it should be emphasized that this method has an inherent bias as it only considers nearby sequences, regardless of genetic distance [31]. Due to this limitation, some researchers have started to propose the use of BCM as a replacement for BM for general analyzes [82]. When the genetic distance threshold is increased in the analyzes (BCM and BIC), variation in the hit rate and specimen identification is observed. This variation is attributed to the presence of species with high intraspecific variation, which makes the assignment of certain sequences difficult [84]. At a threshold of 2% in the BIC method, the sequences of *N. ephratae* and *N. coxipoensis* specimens were categorized as ambiguous due to the low interspecific divergence between these two species

Although it was not possible to find a barcode gap value in this study, the species delimitations using COII showed a high convergence rate with the morphological delimitation (95.83%), indicating that it is a gene capable of delimiting termite species, and the genetic data showed that it can be used as a termite species identification tool. Although it was not possible to obtain all the sequences of the termite species present in the state of Paraíba, the information collected in this study represents a significant advance in the field of taxonomy. This advance is particularly noteworthy because it contributes the genetic data of 15 species to public databases and because 14% of the termite species already known to occur in Brazil have been sequenced here. In addition, this genetic data is another tool that helps in the identification of species. This usefulness is emphasized by the fact that regional genetic data help to improve the accuracy of identifications [84].

## Conclusions

The creation of a genetic data of COII sequences of termites from the state of Paraíba is a significant advance both for taxonomy and for the conservation of Brazilian biodiversity. This initiative is particularly important in a region where there is a lack of studies to understand phylogenetic diversity. In addition, the genetic data provide essential support for conducting biomonitoring through mass sequencing. Our results suggest that the COII gene is effective in delimiting species and is predominantly consistent with morphological identifications. Although it was not possible to identify a DNA barcode gap due to the overlaps found, this limitation did not prevent us from proving the efficiency of COII, as a minimum capacity of 91.50% correct identifications was achieved.

## Supporting information

S1 Table List of investigated species and termites with the corresponding GenBank accession numbers. (ODS)

S2 Table GenBank accession numbers retrieved for subsequent analysis. (ODS)

S3 Table Intraspecific and interspecific divergence for the COII gene of the analyzed species. (ODS)
